# New insights into the characteristics of DRAK2 and its role in apoptosis: From molecular mechanisms to clinically applied potential

**DOI:** 10.3389/fphar.2022.1014508

**Published:** 2022-10-28

**Authors:** Youwei Zheng, Xinchao Li, Lirun Kuang, Yong Wang

**Affiliations:** Department of General Surgery, The Fourth Affiliated Hospital of China Medical University, Shenyang, China

**Keywords:** DRAK2, DAPK, STK17B, apoptosis, targeted therapy

## Abstract

As a member of the death-associated protein kinase (DAPK) family, DAP kinase-associated apoptosis-inducing kinase 2 (DRAK2) performs apoptosis-related functions. Compelling evidence suggests that DRAK2 is involved in regulating the activation of T lymphocytes as well as pancreatic β-cell apoptosis in type I diabetes. In addition, DRAK2 has been shown to be involved in the development of related tumor and non-tumor diseases through a variety of mechanisms, including exacerbation of alcoholic fatty liver disease (NAFLD) through SRSF6-associated RNA selective splicing mechanism, regulation of chronic lymphocytic leukemia and acute myeloid leukemia, and progression of colorectal cancer. This review focuses on the structure, function, and upstream pathways of DRAK2 and discusses the potential and challenges associated with the clinical application of DRAK2-based small-molecule inhibitors, with the aim of advancing DRAK2 research.

## Introduction

Death-associated apoptosis-inducing protein kinase 2 (DRAK2) (Gozuacik and Kimchi, 2006), also known as STK17B, is a serine/threonine protein kinase and a member of the death-associated protein kinase (DAPK) family. As the name implies, DRAK2 is primarily associated with apoptosis, particularly in pancreatic beta cells (Mao et al., 2008; Wang et al., 2017). Several studies have shown that DRAK2 plays an important role in the development of type 1 diabetes (Edwards et al., 2015). The mRNA levels and protein levels of DRAK2 in pancreatic β-cells are rapidly increased in response to inflammatory lymphokine stimulation, ultimately leading to apoptosis of islet β-cells. Another relatively important and currently recognized function of DRAK2 is its involvement in the activation of lymphoid T-cells (Fracchia et al., 2013). One study showed that DRAK2 is a negative regulator of T-cell receptor (TCR) signaling and sets the threshold for T-cell activation through this pathway (Friedrich et al., 2005). Although DRAK2 is a member of the DAPK family, it has been much less studied than other members of the family, such as DAPK1, DAPK2, and DAPK3 (Dai et al., 2016). While DRAK2 shows similar pro-apoptotic functions to the other members (Rennier and Ji, 2013; Benderska and Schneider-Stock, 2014), it is structurally different from DAPK1/2/3 and only shows structural similarity to DRAK1 (Farag and Roh, 2019). DRAK2 began to receive research attention in the 1990s, with the earliest studies reporting its structure and the relationship with lymphoid T-cell activation. Subsequent studies gradually showed that DRAK2 is involved in the development of many cancers, such as acute myeloid leukemia (Ye et al., 2013) and colorectal cancer (CRC). In addition, researchers have also identified an important role of DRAK2 in organ transplant rejection (Gao et al., 2014), which is likely to be an important target for avoiding immune rejection of transplanted organs in the future. More recently, the research focus on DRAK2 has been renewed with studies demonstrating an important link between DRAK2 and non-alcoholic fatty liver disease (NAFLD) (Li et al., 2021). DRAK2 was found to be expressed to varying degrees in patients and mice with different severities of fatty liver, suggesting that it plays a crucial role in metabolic disease.

Current research on DRAK2 is relatively limited, and although DRAK2 has been shown to be involved in many physiological or pathological activities, a large proportion of the underlying specific mechanisms have not been identified. For example, as mentioned above, DRAK2 affects NAFLD, and one study suggested that it may be involved in the development of NAFLD by affecting the splicing mechanism of RNA (Li et al., 2021). Although this study focused on the changes downstream of DRAK2, it did not mention how DRAK2 acts in the early stages of the disease. Similarly, for example, DRAK2 overexpression was found to promote apoptosis in islet β-cells treated with free fatty acid (FFA), but the mechanism by which FFA caused DRAK2 overexpression was not specified.

Phorbol myristate acetate (PMA) has been used by many researchers to induce the expression of DRAK2 in various cells (Kuwahara et al., 2008). PMA can also induce the translocation of DRAK2 from the NIH3T3 cytoplasm to the nucleus, but the mechanism by which PMA induces DRAK2 expression has not been explained. Thus, research on DRAK2 has remained superficial and the mechanisms involved have not been clearly investigated. Nevertheless, research on this topic is ongoing, and in the absence of a detailed summary of the literature on this topic, the accumulating literature has become increasingly confusing. To address these issues, this article reviews the structure, function, upstream pathways, and small-molecule inhibitors of DRAK2 on the basis of previous publications. We hope that the manuscript will provide readers a quick overview of DRAK2.

## The structure of DAPK

Protein kinases are enzymes that catalyze protein phosphorylation. Currently, there are two known types of protein kinases: protein tyrosine kinase (PTK) and serine/threonine protein kinase (STK) (Farag and Roh, 2019). The DAPK family is one of the important STK families and includes five members: DAPK1, DAPK2 (also known as DAPK-related protein 1 [DRP-1]) (Shiloh et al., 2014; Geering, 2015; Yan et al., 2019), DAPK3 (also known as DAP-like kinase [DLK] or zipper-interacting protein kinase [ZIPK]), and DRAK1 and DRAK2 (also known as DARK-related kinase 1 [DRK-1] and DARK-related kinase [DRK-2], respectively). The five members are highly homologous at the N-terminal end of the amino acid sequence and differ at the C-terminal end, which is related to their functions. Among them, DAPK3 has about 83% homology with DAP kinase within the kinase structural domain (Heyerdahl et al., 2010; Cao et al., 2013), while the two most closely related members of the DAPK family, DRAK1 and DRAK2, have 50% homology with DAP kinase within the kinase structural domain. In addition, the DAPK family also belongs to the calmodulin (CaM)-regulated kinase superfamily, but not all five family members contain CaM-regulated regions, with DAPK1 and DAPK2 containing calcium-regulated regions and DAPK3, DRAK1, and DRAK2 not containing these regions.

**FIGURE 1:**
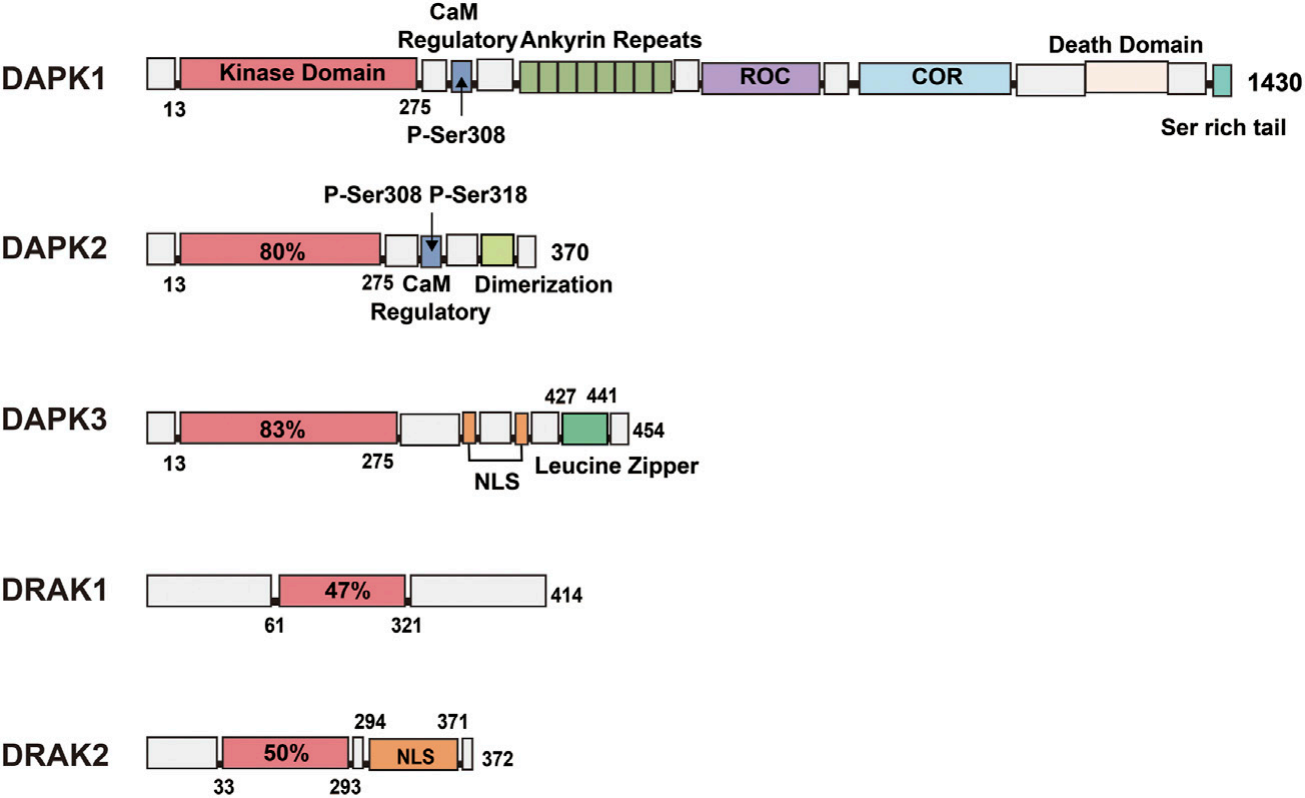
Schematic representation of the kinase structural domains and respective positions of each DAPK family member. DAPK1: In addition to the catalytic kinase structural domain, contains a CaM regulatory region, eight anchor protein repeats, ROCO structural domain, cytoskeleton binding region, death structural domain and a serine-rich C-tail. DAPK2: shares 80% of the catalytic structural domain with DAPK1, and additionally contains a CaM regulatory region and a dimerization element. DAPK3: shares 83% of the catalytic structural domain with DAPK1 and contains a nuclear localization signal (NLS) sequence and a leucine zipper structural domain. DRAK1: shares only 47% of the catalytic structural domain with DAPK1. DRAK2: consists of a catalytic structural domain and a nuclear localization signal.

DAPK1 is the largest member of the family, with a molecular weight of 160 kDa and 1430 amino acids (Nair et al., 2013). In addition to containing a catalytic kinase domain (CD) at the N-terminal (Figure 1), the autoregulatory domain (ARD) next to the CD can exert kinase activity by binding Ca^2+^/CaM through Ser308 in DAPK1 (Temmerman et al., 2013). When Ser308 is phosphorylated, the ARD cannot bind to CaM, leading to inactivation of DAPK1. This process suggests that calcium-activated CaM inhibits catalytic activity by binding to its own regulatory/CaM-binding fragment and the catalytic cleft in this region (Kuczera and Kursula, 2012; Tavares et al., 2017). Although the activation of DAPK1 relies mainly on CaM, even in the absence of CaM, dephosphorylation of DAPK1 by Ser308 can result in low levels of catalytic activity (Simon et al., 2016; Horvath et al., 2021). The structure of DAPK1 also shows eight anchor protein repeats at the right end of the ARD, the ROCO structural domain, the cytoskeleton-binding region, the death structural domain, and a serine-rich C-tail. In addition to containing a catalytic domain 80% homologous to DAPK1, DAPK2 also contains an ARD that binds Ca^2+^/CaM, but when its internal Ser308 is phosphorylated, DAPK2 loses its kinase activity. It contains an additional dimerization element (Wu et al., 2020; Du and Kong, 2021) at its right end (Figure 1), and similar to the inactivation of DAPK1 by Ser308 phosphorylation, Ser318 dephosphorylation can promote dimerization and increase DAPK2 activity (Shoval et al., 2011). In addition, DAPK1 and DAPK2 show similarities in their regulation of kinase activity within the catalytic structural domain and both show the following two aspects: *1*) upon Ser308 dephosphorylation, calcium-activated CaM binds to the self-regulated/CaM-bound fragment pulling the structural domain out of the catalytic cleft; *2*) simultaneously, CaM binding promotes the dephosphorylation of Ser308 (Shoval et al., 2011; Simon et al., 2016), and the dephosphorylated Ser308 increases the DAPK2 activity by promoting dimerization like the dephosphorylated Ser318; therefore, the binding of CaM and the dephosphorylation of Ser308 are reciprocal. In addition, a number of studies have confirmed this role of Ser308 in DAPK1 and DAPK2, replacing all Ser308 in both kinases with Ala or deleting the CaM-binding region in both kinases to produce constitutively active kinases that exhibit more potent killing and non-Ca-dependent catalytic activity (Bialik and Kimchi, 2006; Aziz et al., 2009). In addition to an N-terminal catalytic domain that is 83% homologous to DAPK1, DAPK3 also has a nuclear localization signal (NLS) sequence and a leucine zipper domain at the C-terminus (427–441). Although it does not have the CaM regulatory region of DAPK1 and DAPK2, it has three serine/threonine phosphorylation sites within the CD, Thr180, Thr225, and Thr265 (Figure 1), which may be involved in the regulation of DAPK3 activity (Graves et al., 2005). The remaining two members of the DAPK family are less complex in structure than the first three family members, with DRAK1 (also known as STK17A) showing only an N-(Boosen et al., 2009) terminal catalytic domain and a C-terminal regulatory domain containing seven exons and seven introns.

DRAK2, also known as STK17B, is mainly expressed in developing and mature lymphocytes (Ramos et al., 2008), and to a lesser extent in the liver and pancreas, in addition to the thymus. DRAK2 contains 372 amino acids, consists of an N-terminal catalytic domain and a C-terminal domain responsible for regulating kinase activity (Boosen et al., 2009), possesses several relatively significant phosphorylation sites such as Ser10, Ser12, Ser351 (Fracchia et al., 2013), and is autophosphorylated. It also contains an NLS at the C-terminus (Figure 1), and Ser350 is a phosphorylation site for protein kinase C (PKC)-g, whose phosphorylation can affect the nuclear localization of DRAK2 (Kuwahara et al., 2008). This enzyme can induce apoptosis and regulate cell differentiation, and overexpression of DRAK2 in cell lines can induce apoptosis (Guo et al., 2009; Manivannan et al., 2019).

## Functions of the DAPK

Each member of the DAPK family is associated with apoptosis by name and can cause some degree of damage when overexpressed in cells, such as rounding of the cell shape, blistering of membrane structures, detachment from the extracellular matrix, and formation of autophagic vesicles (Chen et al., 2006; Bialik and Kimchi, 2010; Levin-Salomon et al., 2014). The most important features of apoptosis, namely, cell rounding and membrane blistering, are caused by phosphorylation of Ser19 within DAPK, which further allows kinases to act on the myosin II light chain (MLC) within the cytoskeleton, leading to cell spreading, cell motility, cytoplasmic disintegration, and cell death (Dos Santos et al., 2018; Markwardt et al., 2018). DAPK is required for multiple death signals to induce cell death (Bialik and Kimchi, 2006). For example, DAPK1 is associated with induction of autophagy during endoplasmic reticulum (ER) stress (Gozuacik et al., 2008; Xiang et al., 2020). In addition, DAPK1 is a tumor suppressor that inhibits the transformation of normal cells into abnormal cells in the early stages of tumorigenesis (Martoriati et al., 2005), and inhibits tumor metastasis through its effect on the cytoskeleton (Ivanovska et al., 2014). For example, in tumor cell lines lacking p53, the pro-apoptotic activity of DAPK1 gradually disappears (Silginer et al., 2014; Vitillo et al., 2016); thus, DAPKs generally work together to prevent tumors during tumorigenesis by both promoting apoptosis and inhibiting the migration of tumor cells.

In addition to the abovementioned aspects, DAPK1 is present in high levels in the brain, and some studies have found an association between DAPK1 and neuronal cell death (Fujita and Yamashita, 2014; Shi et al., 2022; Zhang et al., 2022), with deletion of the *DAPK1* gene preventing ischemic neuronal death, and the use of DAPK1 inhibitors producing the same results. Activation of DAPK1 induces Ca^2+^ entry into cells *via* functional NMDA receptors (NR2B subunits) in CNS neurons, which in turn leads to cell death (Tu et al., 2010). Because DAPK2 belongs to the same family as DAPK1, it functions similarly to DAPK1 and can also inhibit tumorigenesis and migration by the two methods mentioned above. For example, DAPK2 levels are reduced in Hodgkin’s lymphoma that initiates methylation, and its introduction into cells can promote apoptosis and inhibit tumor growth (Xiang et al., 2020). In addition to these functions, DAPK2 has been associated with autophagy, oxidative stress in cancer cells, myeloid differentiation, and erythropoiesis (Rizzi et al., 2007; Fang et al., 2008; Ber et al., 2015; Schlegel et al., 2015). DAPK3 is currently thought to have a primary function in regulating apoptosis and smooth muscle contraction (Usui et al., 2014a; Usui et al., 2014b; Komatsu and Ikebe, 2014), and also shows tumor-suppressive, apoptosis-promoting, and autophagic functions. Interestingly, DAPK3 can also regulate myosin by inhibiting MLC phosphatase or regulating the light chain (LC20), which in turn enhances responsiveness to Ca^2+^ and induces smooth muscle contraction (Wang L. et al., 2021). In addition, DAPK3 is also associated with cardiovascular diseases (Chang et al., 2010; Carlson et al., 2018; Zhang et al., 2019), and it can regulate myocardial contraction by phosphorylating the MLC at Ser15. In addition to its role in promoting apoptosis, DRAK1 has been most studied for its association with cervical cancer (Manivannan et al., 2019; Chen and MacDonald, 2022). DRAK1 inhibits the growth and metastasis of advanced cervical cancer cells through two pathways: interfering with the homo-oligomerization of tumor necrosis factor (TNF) receptor-associated factor 6 (TRAF6) and specifically reducing the stability of TRAF6 protein through an autophagy-mediated degradation pathway (Park et al., 2020). At the same time, DRAK1 has also been found to function as a novel negative regulator of the transforming growth factor-β (TGF-β) tumor suppressor signaling pathway. DRAK1 can interrupt the formation of the Smad3/Smad4 complex by binding to Smad3, a process that inhibits TGF-β tumor suppressor signaling in head and neck squamous cell carcinoma (HNSCC) and increases tumor activity (Park et al., 2015). In addition, DRAK1 has also been associated with the development of SLE (da Silva Fonseca et al., 2013) and testicular cancer (Mao et al., 2011).

The functional aspects of DRAK2 have been evaluated in some studies, but most of them focus on T-cell activation, islet cell function, *etc.* For example, DRAK2 is a negative regulator of TCR signaling and sets the threshold for T-cell activation through this pathway (Ramos et al., 2008),. Moreover, inhibition of DRAK2 expression can prevent islet β-cell apoptosis (Wang et al., 2017). DRAK2 has been recently suggested to be involved in the development of NAFLD by inhibiting the phosphorylation of serine-arginine-containing splicing factor 6 (SRSF6) by SRSF protein kinase 1 (SRPK1) through binding to SRSF6 (Li et al., 2021). We found that this enzyme plays an important role in apoptosis, immunity, and metabolism. This article compiles and summarizes the existing literature on DRAK2. The upstream pathways and small-molecule inhibitors of DRAK2 are described separately below.

## The upstream pathways of DRAK2

### MYB

**FIGURE 2:**
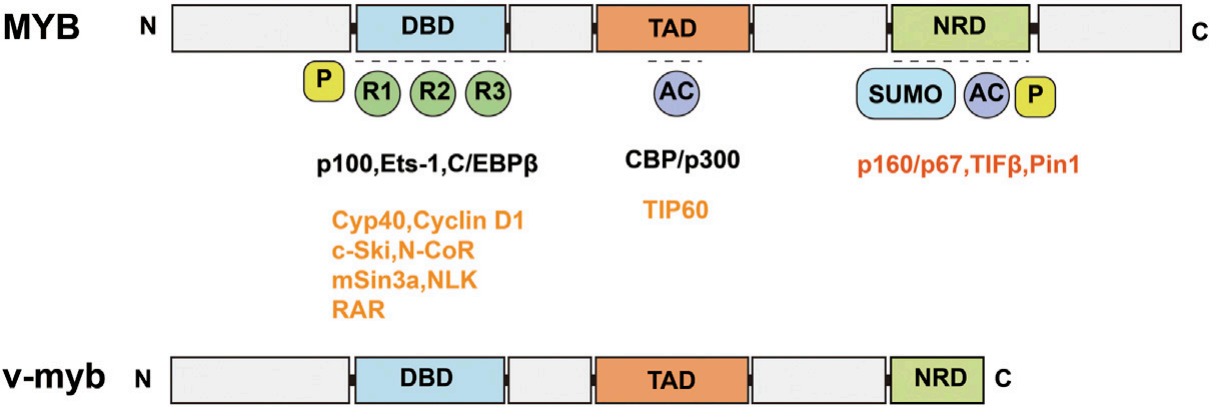
Schematic structure of MYB and v-myb. MYB consists of an N-terminal DNA binding domain (DBD), a C-terminal negative regulatory domain (NRD) and a central trans-activation domain (TAD). v-myb is derived from MYB mutations.

MYB is a segment of the proto-oncogene that encodes a protein with three HTH DNA-binding structural domains. This structural domain has a transcriptional regulatory role. MYB is an important transcription factor that is inextricably linked to hematopoietic function (Greig et al., 2008; Shah et al., 2021). MYB gene family members include MYB, MYBL1, and MYBL2 (Ciciro and Sala, 2021). *MYB* genes contain an N-terminal DNA-binding domain (DBD), a C-terminal negative regulatory domain (NRD) and a central trans-activation domain (TAD) (Figure 2) (Ko et al., 2008); v-myb is derived from mutations in the *MYB* gene and can cause severe acute myeloid leukemia in vertebrates (Weng et al., 2018; Wang et al., 2020; Smeenk et al., 2021). DRAK2 plays an important role in v-myb-mediated acute myeloid leukemia (Ye et al., 2013), and CHIP indicates that MYB binds to a conserved element upstream of the DRAK2 transcription start site. Knockdown of the *MYB* gene using MYB shRNA in U937 cells can induce apoptosis in U937 cells by stimulating DRAK2 expression and downstream caspase-9 activity. In addition, in a clinical study of 22 AML patients (Lee et al., 2006), expression profiling of bone marrow samples revealed MYB upregulation and DRAK2 downregulation in seven patients. In other related studies, DRAK2 expression was also found to be twice as high after inhibition of the *MYB* gene using MYB shRNA in an AML mouse model (Zuber et al., 2011). In conclusion, all of the above studies demonstrated that DRAK2 is a downstream target gene of MYB.

### Protein kinase D

**FIGURE 3:**
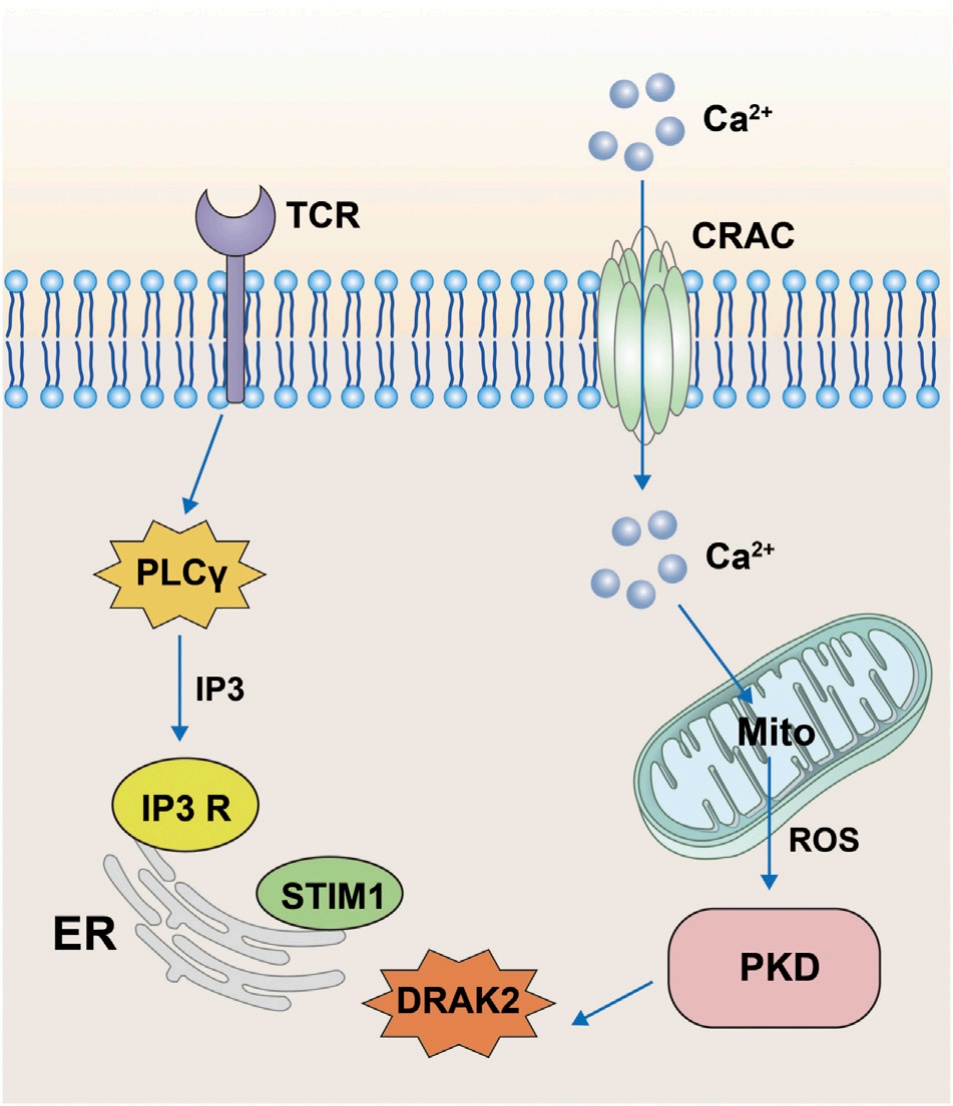
Pathway diagram of DRAK2 involvement in T-cell activation. When the T-cell receptor receives the signal, it acts on the receptors on the surface of the endoplasmic reticulum *via* IP3 generated by PLCγ, resulting in the release of Ca ions from the endoplasmic reticulum. When STIM1 senses Ca ion deficiency, extracellular Ca ions will flow inward through CRAC channels. The inward flow of Ca ions stimulates the mitochondria to produce large amounts of ROS, which then influence the expression of DRAK2 through the PKD signaling pathway.

Protein kinase D (PKD) belongs to the serine/threonine kinase family and the Ca^++^-calmodulin-dependent protein kinase (CaMK) family. PKD contains three isoforms: PKD1, PKD2, and PKD3, with PKD1 being called PKDμ in the earliest study in 1994 (Bush and McKinsey, 2009) and PKD3 being called PKDv (Papazyan et al., 2008). All three isoforms perform important roles in cellular functions, including intracellular vesicle transport and maintenance of Golgi function. PKD has been shown to be involved in the activation of T-cells and its main pathway is through the regulation of DRAK2 activity (Newton et al., 2011). T-cell activation is mainly associated with Ca^2+^ influx; however, when PKD is knocked out, it strongly blocks the TCR signaling pathway and prevents the activation of DRAK2. Moreover, activation of DRAK2 is inhibited by the PKD small-molecule inhibitor Gö6976. In one specific mechanism, when the TCR is stimulated by certain signals, it produces IP3 through activation of phospholipase Cγ (PLCγ), which acts on the IP3 receptor on the ER, causing release of Ca ions from the ER. If the stromal sympathetic molecule 1 (STIM1) on the ER senses Ca ion insufficiency, it stimulates cell surface calcium release to activate calcium channel regulatory molecule (ORAI1) into the CRAC channel, which causes the inward flow of extracellular Ca ions. Ca ions can stimulate mitochondria to produce large amounts of reactive oxygen species (ROS) under certain circumstances (Schieber and Chandel, 2014; Zorov et al., 2014; Hamilton et al., 2020), and the mitochondrial production of ROS activates DRAK2 by stimulating PKD. DRAK2 acts on STIM1 to regulate the concentration of Ca ions, which in turn regulates T-cell differentiation (Figure 3). In addition, the study also confirmed that DRAK2 is a direct substrate of PKD (Newton et al., 2011). The team found reduced basal autophosphorylation levels of DRAK2 and greatly reduced PMA-induced DRAK2 autophosphorylation when using PKD mutants (KD-PKD1; PKD1-K612W) co-expressed with DRAK2. Moreover, infection of JurkaT-cells with PKD2-expressing shRNA revealed that the DRAK2 activation induced by anti-CD3 cross-linking or toxic carotenoids was greatly reduced by PKD2 knockdown. In conclusion, PKD has been shown to be a substrate for DRAK2 by a number of different methods.

### Cyclooxygenase 2

Cyclooxygenase 2 (COX-2) is an inducible enzyme regulated by many cytokines, such as interleukin (IL)-1β, IL-6, or TNFα (Chan et al., 2019), and is primarily responsible for prostaglandin (PG) biosynthesis (Karpisheh et al., 2019; Frejborg et al., 2020; Ochiai et al., 2022). COX-2 expression in normal cells is rare and almost negligible (Gurram et al., 2018), and its frequent appearance in cancer is considered a marker for cancer detection (Hashemi Goradel et al., 2019). Some studies have shown that COX-2 expression is increased in most CRC patients, suggesting that COX-2 is closely related to CRC (Wang and Dubois, 2010; Su et al., 2016; Dagallier et al., 2021). Interestingly, when the COX-2 selective inhibitor rofecoxib was administered to CRC patients, the DRAK2 level in tumor cells increased 2.5-fold. In addition, inhibition of COX-2 activity in HCA7 cells also enhanced the expression of DRAK2 (Doherty et al., 2009). The COX-2 transcriptional level in CRC patients was found to be 2.4 times higher than normal, while the opposite was true for DRAK2 expression. Other studies have also highlighted the involvement of COX-2 in the negative regulation of DRAK2. Overexpression of the pro-apoptotic gene DRAK2 was also present in studies in COX-2−/− mice treated with adriamycin Dox (Neilan et al., 2006). Interestingly, COX-2 has also been shown to regulate T-cell activation (Li Q. et al., 2022). Therefore, in combination with the abovementioned involvement of DRAK2 in T-cell activation, we speculate that COX-2 and DRAK2 show some similarities or crossover in the pathways that regulate T-cell activation.

### TGF-β

**FIGURE 4:**
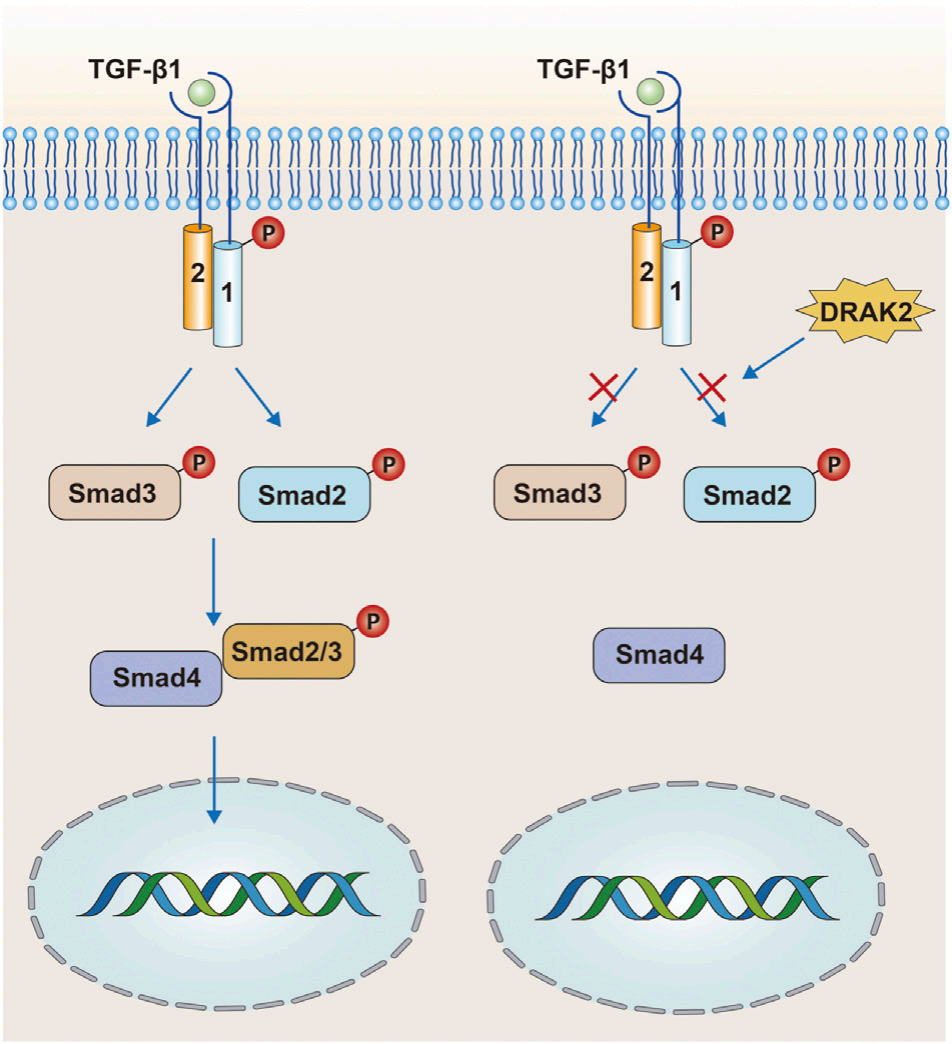
In the tumor environment, DRAK2 can promote tumor growth by blocking TGF-β signaling. DRAK2 acts mainly by blocking phosphorylated Smad2/3 proteins from binding to Smad4 and thus blocking the entry of the complex into the nucleus. However, later studies have shown that DRAK2 is not involved in this pathway.

TGF-β is a multicellular functional factor involved in the regulation of multiple intracellular activities with three ligands: TGF-β1, TGF-β2, and TGF-β3 (Rubtsov and Rudensky, 2007). In the classical TGF-β signaling, TGF-β binds to the type II TGF-β receptor (TβRII) on the cell membrane, followed by recruitment and phosphorylation of the type I TGF-β receptor (TβRⅠ). The phosphorylated TβRⅠ then acts through the Smad protein, and the heterodimeric complex composed of TβRⅡ and phosphorylated TβRⅠ in turn phosphorylates Smad2 and Smad3 downstream, while the phosphorylated Smad2/3 binds to Smad4 (Figure 4) and finally enters the nucleus to act. However, some studies have confirmed that DRAK2 can act as an antagonist of TGF-β signaling induced by TGF-β1 (Yang et al., 2012). In this process, DRAK2 can bind specifically to TβRⅠ, thus blocking the activation of Smad2/3 by phosphorylated TβRⅠ, while the unphosphorylated Smad2/3 cannot bind to Smad4 and thus cannot enter the nucleus for regulation. Another study published in *The Lancet* found that DRAK2 was abundantly expressed in breast cancer cells and that knockdown of DRAK2 in breast cancer cells enhanced TGF-β signaling (Wang et al., 2005). Therefore, we speculate that in breast cancer cells, DRAK2 is likely to promote tumor growth by blocking TGF-β signaling. However, the ability of DRAK2 to block TGF-β1-induced TGF-β signaling has been questioned (Harris and McGargill, 2015). In that study, T-cells were isolated from wild-type and Drak2^−/−^ mice, and TGF-β signaling was not enhanced in Drak2^−/−^ T-cells after an exogenous increase in TGF-β1 and did not differ significantly from the wild-type T-cells. There are several possible reasons for this phenomenon: *1*) The study in which DRAK2 blocked TGF-β1-induced TGF-β signaling was mainly performed in cancer cells rather than in the normal physiological state, while the latter study was performed in normal T-cells. Thus, the differences in results may be attributable to differences in the function or mode of action of DRAK2 in the normal physiological state and in the cancer setting. *2*) Although enhanced TGF-β signaling was not found in Drak2−/−T-cells, the level of Smad7, a negative regulator of TGF-β signaling, was higher than that in wild-type T-cells (Itoh and ten Dijke, 2007). This phenomenon may be due to the fact that Drak2 −/− T-cells compensate for the absence of DRAK2 through other alternative pathways, which in turn affects TGF-β signaling. The mechanisms involved in the functioning of DRAK2 in TGF-β signaling in normal *versus* abnormal environments, *in vivo* and *in vitro*, are unclear and need to be further investigated.

### Interferon-gamma, TNF-α and IL-1β

Interferon-gamma (IFN-γ), TNF-α, and IL-1β are closely associated with the development of type I diabetes (Arif et al., 2021; Janssen et al., 2021; Sims et al., 2021). One of the main reasons for the development of type I diabetes is that cytokines such as IFN-γ, TNF-α, and IL-1β produced by immune cells break down the islet beta cells. Curiously, one study found that only prolonged treatment of cells with IL-1β + IFN-γ and/or TNF-α resulted in islet β-cell dysfunction and eventual apoptosis, but individual cytokines did not produce similar results (Kim et al., 2007). However, one study found that the effects of IFN-γ, TNF-α, and IL-1β on pancreatic islet beta cells were associated with the activation of DRAK2 (Mao et al., 2009). When pancreatic β-cells were treated with IFN-γ plus IL-1β or TNF-α plus IL-1β, they were able to increase the mRNA expression of DRAK2 and induce apoptosis. Interestingly, when pancreatic β-cells were separately treated with the three abovementioned cytokines, they failed to increase the expression of DRAK2. This phenomenon coincides with our previous mention of the inability of the three cytokines to trigger islet β-cell apoptosis when used alone. The destruction of islet β-cells by IFN-γ plus IL-1β is mainly mediated by NO (Cnop et al., 2005), and this process can be blocked by inducible NO synthase (iNOS) inhibitors, which also inhibit the enhancement of DRAK2. A reduction in the number of apoptotic cells could be observed after DRAK2 knockdown, blocking the apoptosis signal mediated by caspase-9 (Mao et al., 2009). Thus, DRAK2 is also involved in this process here and acts downstream of iNOS and upstream of caspase-9.

### NO and iNOS

**FIGURE 5:**
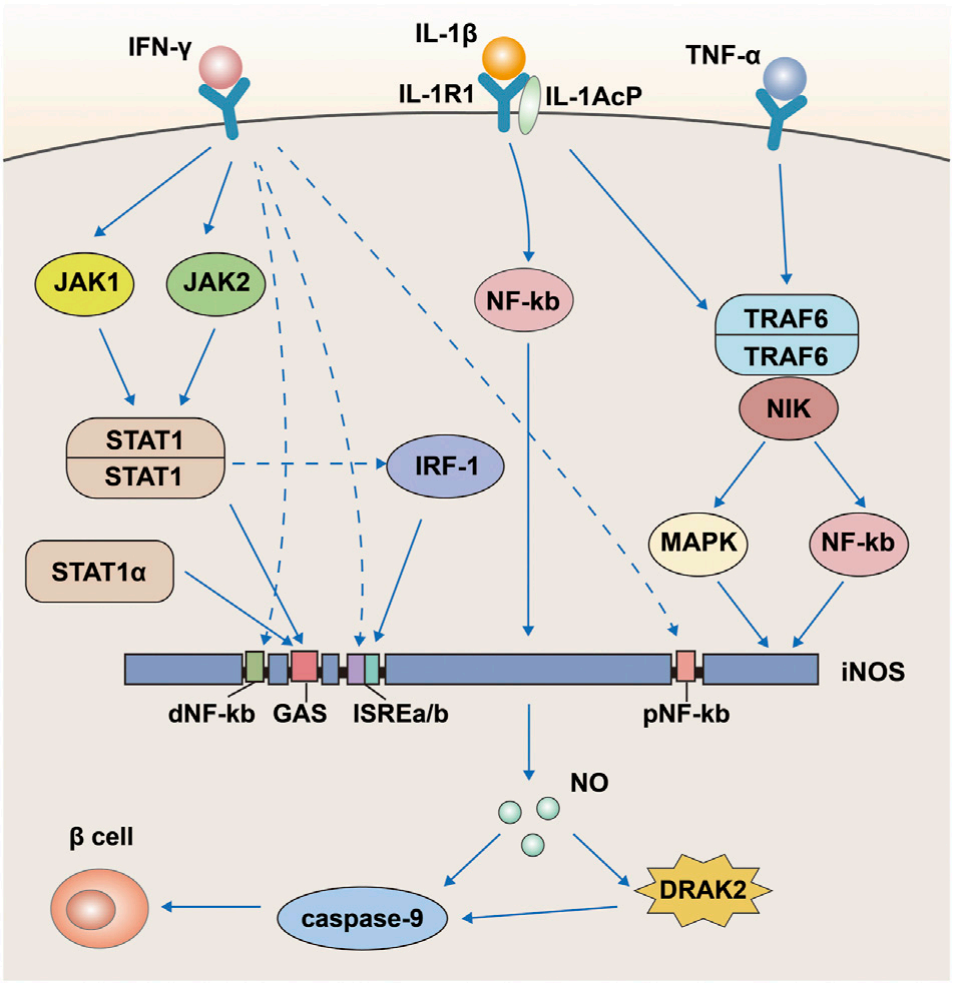
A pathway map of cytokines such as IFN-γ, TNF-α and IL-1β that promote apoptosis in pancreatic β-cells *via* DRAK2.

We mentioned above that NO and iNOS are involved in the effects of IFN-γ, TNF-α, and IL-1β on pancreatic β-cells, and that DRAK2 is also involved in between. Some studies have identified the specific mechanisms involved in these effects (Reddy et al., 2021). This process is associated with the activation of the nuclear factor (NF)-kb signaling pathway by IL-1β and the expression of iNOS (Figure 5). The iNOS promoter has two regions, proximal and distal, containing DNA-binding elements for different transcription factors. The proximal region includes an NF-kb and the distal region includes another NF-kb, an IFN-γ activation site (GAS), and two IFN stimulatory response elements (ISRES) right next to each other. First, IL-1β can stimulate both NF-kb sites proximal and distal to the iNOS promoter, of which the site at the far end is the primary site; however, IL-1β alone is not sufficient to activate iNOS as a single cytokine. IFN-γ simultaneously activates tyrosine kinases JAK1 and JAK2 *via* surface receptors, followed by dimerization of the phosphorylated transcription factor STAT-1 to bind to the GAS (Neagu and Constantin, 2021). In addition, lipopolysaccharide in macrophages can directly induce the STAT-1 isoform STAT-1α and bind to the GAS (Salim et al., 2016). Besides, STAT can also increase the expression of iNOS by inducing the transcription factor IRF-1 (Sudhakar et al., 2013), and the increased expression of iNOS will eventually produce more NO to damage islet β-cells. Thus, in the apoptosis of pancreatic β-cells, DRAK2 acts downstream of NO and upstream of caspase-9. TNF-α likewise assists IL-1β in inducing apoptosis in pancreatic islet β-cells (Kaminitz et al., 2017; Xie et al., 2018; Quattrin et al., 2020; Perdigoto et al., 2022). When TNF-α binds to its receptor, it induces the production of TRAF6, while the IL-1/IL-1R1/IL-1AcP complex induced by IL-1β simultaneously binds to TRAF6 and forms a new complex (Li L. et al., 2022), stimulating iNOS expression *via* the mitogen-activated protein kinase signaling pathway (MAPK) (Xiong et al., 2017; Sato et al., 2018). In addition, TRAF6 is also involved in NF-xB activation *via* NF-xB-inducible kinase (NIK) (Li and Wang, 2022). In conclusion, we learned from the above analysis that IFN-γ, TNF-α, IL-1β, NO, and iNOS are all upstream of DRAK2 and can affect the expression of DRAK2 through different pathways.

### ROS and free fatty acids

ROS and free fatty acids (FFA) are also upstream of DRAK2, and as we mentioned above, ROS can regulate DRAK2 through PKD during T-cell activation. In addition to the process of T-cell activation, ROS can also exert apoptotic effects through PKD in other environments (Cobbaut and Van Lint, 2018). For example, under oxidative stress, PKD1 in cancer cells can regulate NF-kb through the IKK complex (Yuan et al., 2022). Moreover, in MCF7 cells, H_2_O_2_ activates NIK and phosphorylates IKKα *via* induction, followed by activation of NF-kb *via* a non-classical pathway (Li and Engelhardt, 2006). This process then intersects with the pathway through which IFN-γ, TNF-α, and IL-1β regulate DRAK2 *via* NF-kb. FFA has also been shown to induce DRAK2 under certain conditions (Mao et al., 2008; Lan et al., 2018; Li et al., 2021; Wu and Kapfhammer, 2021). In primary mouse hepatocytes, DRAK2 expression at both the protein level and the RNA level increased with increasing concentrations of palmitic acid (PA) and with increasing duration of action (Li et al., 2021). The study also confirmed that the expression of DRAK2 *in vivo* was associated with the development of NAFLD.

## DRAK2-based targeted therapy

DRAK2 is associated with the development of several diseases, such as NAFLD, diabetes mellitus (McGargill et al., 2008), and breast cancer. In addition, it is also involved in important processes such as autophagy and T-cell differentiation. Inhibition of DRAK2 expression can avoid a variety of pathological mechanisms and maintain healthy homeostasis. In addition, DRAK2 protein is mainly expressed in lymphoid organs, mostly in B cells, but also in higher amounts in T-cells and not in natural killer (NK) cells, macrophages, or dendritic cells. One study found that when DRAK2 expression was inhibited, mice develop resistance to a T-cell-mediated autoimmune disease—experimental autoimmune encephalomyelitis (EAE) (McGargill et al., 2008; Ramos et al., 2008), and it is also resistant to type I diabetes. Allogeneic rejection has also been recently shown to involve DRAK2 signaling, and inhibition of DRAK2 may maintain graft activity in the long term (Weist et al., 2012). On the basis of these findings, DRAK2 is likely to be a potential drug target for the treatment of autoimmune diseases and the prevention of graft rejection after organ transplantation. Thus, the presence of small-molecule inhibitors of DRAK2 is particularly important. but research on inhibitors of DRAK2 is currently very limited. Therefore, we have presented a compilation of previously published data on small-molecule inhibitors in the literature.

SC82510 was shown to inhibit DRAK2 at very low concentrations (1 nM) and to promote neuronal differentiation and axonal branch growth in pheochromocytoma (PC12) cells; moreover, this process was enhanced by the addition of neuronal growth factor (FGF-2) (Marvaldi et al., 2014). The team first used KINOMEscan^™^ to determine the binding constants of different compounds to 442 eukaryotic kinases and screened two classes of compounds, after which they tested all compounds for inhibition of DRAK2. The results showed that SC84458 has a high DRAK2 inhibitory activity, but its specificity is poor and it also shows inhibitory effects on other kinases. However, SC82510 is characterized by low activity and high specificity, inhibiting only DRAK1, DRAK2, and RPSK2, and it was able to induce neuronal differentiation of PC12 cells at very low concentrations (1/5 nM). However, the chemical structure of SC82510 was not shown in the article.

**FIGURE 6:**
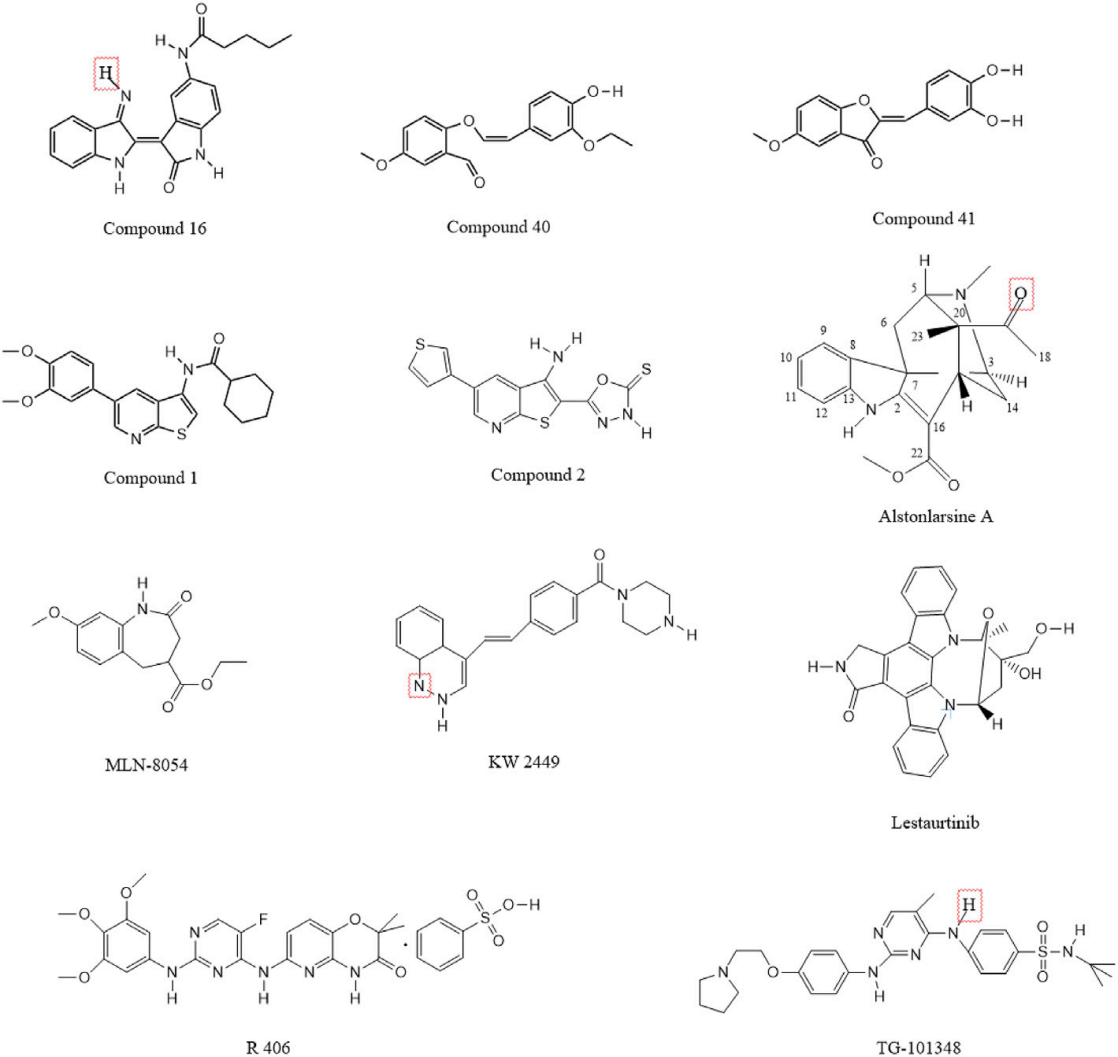
Chemical structure formula of DRAK2 small molecule inhibitor.

Indirubin derivatives were identified as novel DRAK2 small-molecule inhibitors in 2016 (Jung et al., 2016); these derivatives are the main ingredients of two Chinese herbal medicines, Ginseng and Deer Antler Pills and Qing Dai, which have been used to treat chronic granulocytic leukemia in China (Wang et al., 2014; Blazevic et al., 2015; Wang H. et al., 2021). In addition to its anticancer properties, indirubin has also been shown to be effective against diseases such as psoriasis (Hsieh et al., 2012; Sun et al., 2021), Alzheimer’s disease (Chen et al., 2017; Du et al., 2018), and autoimmunity. Although monomeric indirubin shows disadvantages such as poor water solubility and poor drug metabolism kinetics (Wang H. et al., 2021)., but because of its anticancer properties, researchers have attempted to develop derivatives based on indirubin to increase the efficacy and improve the metabolic dynamics of the drug and reduce the original limitations. One team used high-throughput screening to show that indirubin derivatives could act as novel inhibitors of DRAK2. The team first screened 11,000 compounds by an *in vitro* kinase assay using recombinant DRAK2 protein as the zymogen, and finally identified 41 compounds with 70% inhibition effect and strong activity. These compounds were then subjected to high-throughput screening, and those with activity were found to be indirubin or indirubin-3′-monoxime derivatives. A subsequent series of processes yielded 33 compounds, of which compounds 15–19 significantly increased the inhibition of DRAK2 and compounds 22–33 showed only moderate inhibition (detailed information can be found in the corresponding study (Jung et al., 2016)). Among these, compound 16 showed the highest specificity and inhibited DRAK2 in an ATP-competitive manner (Figure 6). This study also demonstrated that 5-N-acyl indirubin-3′-monoxime compounds are novel DRAK2 inhibitors.

Benzofuran-3(2H)-one derivatives have been reported to act as inhibitors of DRAK2 to avoid islet β-cell apoptosis (Wang et al., 2017). The team first identified 2-(3,4-dihydroxybenzylidenebenzofuran-3(2 H)-one as a moderate DRAK2 inhibitor with a half-inhibitory concentration (IC_50_) of 3.15 µM by high-throughput screening. Subsequently, a constitutive relationship (SAR) study was performed, and a total of 36 compounds were synthesized; the addition of methoxy to the 5-, 6-, and 7-positions of benzofuran-3(2H)-one was found to increase the activity of the compounds. The most effective of these were compounds 40 and 41 (Figure 6), which also showed dose-dependent protective effects on pancreatic β-cells from PA-induced apoptosis in the glucose-stimulated insulin secretion assay (GSIS).

A thieno[2,3-b]pyridine derivative was identified as an inhibitor of DRAK2 in 2014 (Gao et al., 2014). However, it also lacked selectivity and has the same inhibitory effect on DRAK1 with an IC_50_ of 0.82 µM. The team first used the KINOMEscan^™^ kinase platform for screening their proprietary compound library, and screened 150 representative compounds as potential ligands for DRAK2 in the DiscoverX binding assay. Finally, a compound based on an isothiazolo[5,4-b]pyridine scaffold was found with a Kd value of 1.6 µM. Subsequently, the isothiazolo[5,4b]pyridine derivative was used as the starting point to generate thieno[2,3-b]pyridine derivatives by binding to the scaffold-jumping method, which was experimentally shown to yield strong binding to DRAK2 (Kd = 9 nM). In general, the process starts with an isothiazolo[5,4-b]pyridine based hit compound with weak affinity for DRAK2, and its substituents are systematically altered to obtain compounds without inhibitory activity but with a binding affinity of 0.5 µM. The scaffold-hopping strategy subsequently revealed that the thieno[2,3-b]pyrazine derivative could serve as an effective ligand for DRAK2. However, the thieno[2,3-b]pyrazine derivative, while having no inhibitory activity against DAPK1, DAPK2 and DAPK3, was not selective for DRAK1 and DRAK2. Therefore, the thieno[2,3-b]pyrazine derivative was considered to be a dual inhibitor of DRAK1 and DRAK2.

Compound 1 (Figure 6) could inhibit the activity of DRAK2 (Farag and Roh, 2019). The compound was mainly obtained as 5-arylthieno[2,3-b]pyridine as a scaffold and by the scaffold-jumping method. Its IC_50_ value for DRAK2 was 0.86 µM, and the Kd value was 9 nM, indicating high specificity but slightly lower inhibitory activity. It could be the starting point for the synthesis of highly selective DRAK2 inhibitors. It also showed an inhibitory effect on DRAK1 with an IC_50_ value of 2.25 µM. The team obtained a series of 5-arylthieno[2,3-b]pyridines by performing some substitution patterns on compound 1, with the most potent compound 2 (Figure 6) having an IC_50_ value of 29 nM and a strong binding affinity (Kd = 0.008 µM) (Leonczak et al., 2014). However, it was a non-selective DRAK2 inhibitor with poor specificity and inhibited DAPK1, DAPK2, DAPK3, and DRAK1, with Kd values of 54 and 99 nM for DAPK1 and DRAK1, respectively.

In addition to some of the small-molecule inhibitors mentioned above, other substances also show DRAK2-inhibiting effects, but they have received less attention in the literature. Oximes have been shown to inhibit DRAK2 (Schepetkin et al., 2021), while 2-benzylidenebenzofuran-3-one was shown to inhibit DRAK2 activity. Drugs such as nintedanib, abemaciclib, and baricitinib can also inhibit its activity, of which nintedanib and abemaciclib were approved by the FDA in 2014 and 2017, respectively. Nintedanib is an intracellular inhibitor of tyrosine kinases that inhibits the processes involved in the progression of pulmonary fibrosis (Flaherty et al., 2019). Although not selective for DAPK inhibition, the affinity of nintedanib for DRAK2 was much higher than that for DAPK2/3, with a K_d_ of 3.2 and 2.1 nM for DAPK2/3 and 110/670 nM for DRAK1/2, respectively. Abemaciclib is an oral, sequentially administered CDK4/6 inhibitor approved for HR+, HER2-advanced breast cancer (ABC) (Goetz et al., 2017; Johnston et al., 2020). However, it is also not selective and is less effective in inhibiting DRAK than DAPK1/2/3 at the same concentration. Baricitinib is an oral, reversible inhibitor of the Janus kinases JAK1 and JAK2 and may have therapeutic value for patients with rheumatoid arthritis. In addition to treating rheumatoid arthritis (Hu et al., 2022; Roskoski, 2022), it has been also shown to inhibit DAPKs, with the percentage of inhibition of DRAK1 and DRAK2 being 99.5% and 98.6%, respectively. In addition to the abovementioned drugs, ISIS Pharmaceutical company also developed antisense oligonucleotides that can inhibit DRAK2. Moreover, Bennett and Dobie in 2011 investigated the selectivity of 72 known kinase inhibitors for 442 kinases *in vivo*. Five compounds, including KW2449, lestaurtinib, MLN-8054, R406, and TG-101348 (Figure 6)**,** were found to have strong inhibitory effects on DRAK2. However, they shared a common disadvantage with the drugs mentioned above, which is that they are not selective. In addition, one study confirmed that Alstonlarsine A (Figure 6), one of the four indole alkaloids isolated from *Alstonia scholaris*, showed moderate inhibitory activity against DRAK2 with an IC_50_ value of 11.65 ± 0.63 μΜ (Zhu et al., 2019). All of the above small molecule inhibitors have shown varying degrees of inhibition of DRAK2 and have some potential for the treatment of related diseases, and are believed to be able to be used in the clinic in the near future.

## Conclusion

Research on DRAK2 has never ceased, moving from initial studies of DRAK2 structure to studies of its function (Chen and MacDonald, 2022), including the discovery of the first important function of DRAK2, i.e., its ability to regulate T-cell activation *via* Ca ions (Friedrich et al., 2005) to the discovery that DRAK2 plays an important role in the immune system (Schaumburg et al., 2007; McGargill et al., 2008). Deletion or low expression of DRAK2 significantly increases resistance to autoimmune diseases. In addition, recent studies have found a correlation between DRAK2 and tumorigenesis in diseases such as chronic lymphocytic leukemia, acute myeloid leukemia (Ye et al., 2013), colorectal cancer, and cutaneous T-cell lymphoma (CTCL) (Hartmann et al., 2008). In the field of drug research, there has been ongoing development of small-molecule inhibitors of DRAK2. Since the discovery that DRAK2 is involved in the development of many cancers, numerous researchers have aspired to hinder disease progression with small-molecule inhibitors of DRAK2, such as the compounds mentioned above.

Notably, recent studies have found a strong link between DRAK2 and the development of NAFLD. This is the first study to show that DRAK2 is associated with a typical metabolic disease. NAFLD (Byrne and Targher, 2015; Friedman et al., 2018; Younossi et al., 2018) is a disease of the liver characterized by hepatic steatosis after excluding other known causes (e.g., high alcohol intake, viral infections, *etc.*), which can further progress to hepatitis, cirrhosis, and even liver cancer. The team demonstrated that DRAK2 exacerbates NAFLD through an SRSF6-related RNA alternative splicing mechanism. When DRAK2 is overexpressed, the kinase binds to SRSF6, resulting in its inability to be phosphorylated by SRPK1. This prevents the complex from entering the nucleus and participating in the splicing process of genes involved in mitochondrial function, thus affecting mitochondrial function and causing NAFLD. However, the study focused on the mechanism by which DRAK2 contributes to the development of NAFLD, so what causes the overexpression of DRAK2 *in vivo*?

As mentioned above in relation to the pathways upstream of DRAK2, all of these pathways can stimulate DRAK2 expression under certain conditions. However, the majority of patients with NAFLD are obese (Fan et al., 2017)., and most obese patients show relatively strong levels of oxidative stress and low levels of inflammation (Karam et al., 2017; Perez-Torres et al., 2021). In combination with our previous description, this finding suggests that obese patients are at high risk of NAFLD because they are prone to oxidative stress and inflammation in the body. The ROS produced by oxidative stress and inflammation leads to high expression of DRAK2 *via* PKD1/2/3. Moreover, overexpression of DRAK2 can damage mitochondrial function and lead to NAFLD, which can eventually further exacerbate oxidative stress and inflammation. Although DRAK2 undoubtedly plays an extremely important role in the early stages of NAFLD development, because of the limitations of research techniques and challenges in clinical application, the mechanisms underlying the promotion of DRAK2 expression by ROS remain to be investigated.
